# Idiopathic omental hemorrhage: a case report and review of the literature

**DOI:** 10.1186/s13256-023-04112-8

**Published:** 2023-08-28

**Authors:** Fatemeh Rashidi, Alireza Samimiat, Niloofar Jafarimehrabady, Reza Hajebi

**Affiliations:** 1 School of Medicine, Tehran University of Medical Sciences, Tehran, 1417613151 Iran; 2 Department of Surgery, Tehran University of Medical Sciences, Tehran, 1417613151 Iran; 3 Department of Clinical-Surgical, Diagnostic and Pediatric Sciences, University of Pavia, 27100 Pavia, Italy; 4 Department of Surgery, Sina Hospital, Tehran University of Medical Sciences, Tehran, 1417613151 Iran

**Keywords:** Omental bleeding, Diagnosis, Computed tomography, Surgery

## Abstract

**Background:**

A spontaneous rupture of an omental vessel can cause severe intraabdominal hemorrhage. We present a case of idiopathic omental hemorrhage caused by a vascular malformation. The literature is systematically reviewed.

**Case presentation:**

A 65-year-old Iranian man was admitted to the emergency department for 10 days with abdominal pain. His medical history was not significant. Fever, vomiting, nausea, or anorexia were not reported. However, he was suffering from diaphoresis and malaise at the time. He did not smoke or drink alcohol. During physical examination, blood pressure was 82/60 mmHg with a temperature of 36.6 °C; heart rate was 96 beats/minute and respiratory rate was 18 breaths per/minute. An abdominal examination revealed mild tenderness in the periumblical. The focused assessment with sonography in trauma examination yielded positive results. The complete blood count showed 14 × 10^3^/mcL of white blood cells and 185 × 10^3^/mcL of platelets. The hemoglobin value was 6.7 g/L at admission. To stabilize the patient’s condition, a unit of packed cell was administered. A double contrast enhancement abdominal computer tomography was performed, which revealed a massive hemoperitoneum. Subsequently, an exploratory laparoscopy was performed to search for the responsible pathology. But it was not successful. The surgical plan was changed to laparotomy. The hemorrhage source was not found during laparotomy. Observation revealed a massive hemoperitoneum originating in the omental vessels. A portion of the omentum located on the greater omentum at the greater curve was removed. Based on the pathological examination of the extracted tissue, vascular malformations were identified. The patient recovered uneventfully and was discharged from the hospital 7 days after surgery. Previous reports assessing idiopathic omental bleeding were systematically reviewed. A total of 14 hits were identified in PubMed and Scopus from 2015 to November 2022 for idiopathic omental bleeding.

**Conclusion:**

Presence of positive focused assessment with sonography in trauma, abdominal pain, imaging evidence of fluid accumulation, and a reduction in hemoglobin levels collectively indicate the likelihood of arteriovenous malformation occurrence. The treatment options include surgical intervention and transcatheter arterial embolization. Surgical intervention is recommended for subjects with hemodynamic instability, persistent hypotension and those whose diagnosis is unconfirmed.

## Introduction

Idiopathic spontaneous intraperitoneal hemorrhage occurs when intraabdominal vessels rupture without an identifiable underlying cause [1].

Omental bleeding may occur due to trauma-associated injury and irritation, neoplasia [2], arterial aneurysm rupture [3], and anticoagulant treatment [4].

Due to their rarity, therapeutic management guidelines are not yet established. We present a case of idiopathic omental hemorrhage caused by a vascular malformation. A systematic review of previous reports was also conducted.

## Case presentation

**Fig. 1:**
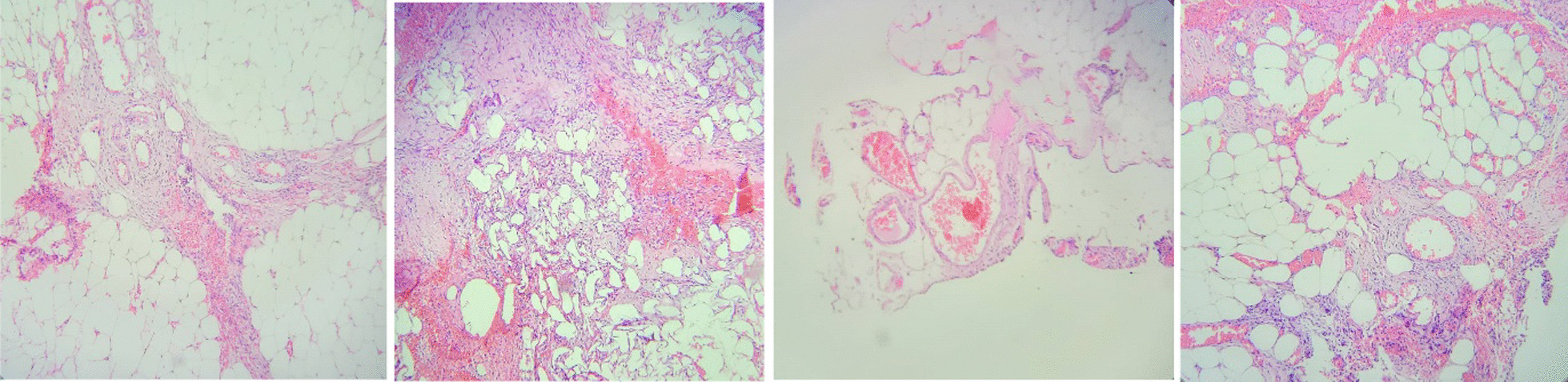
Abnormally dilated blood vessels, an arteriovenous malformation (AVM)

**Fig. 2:**
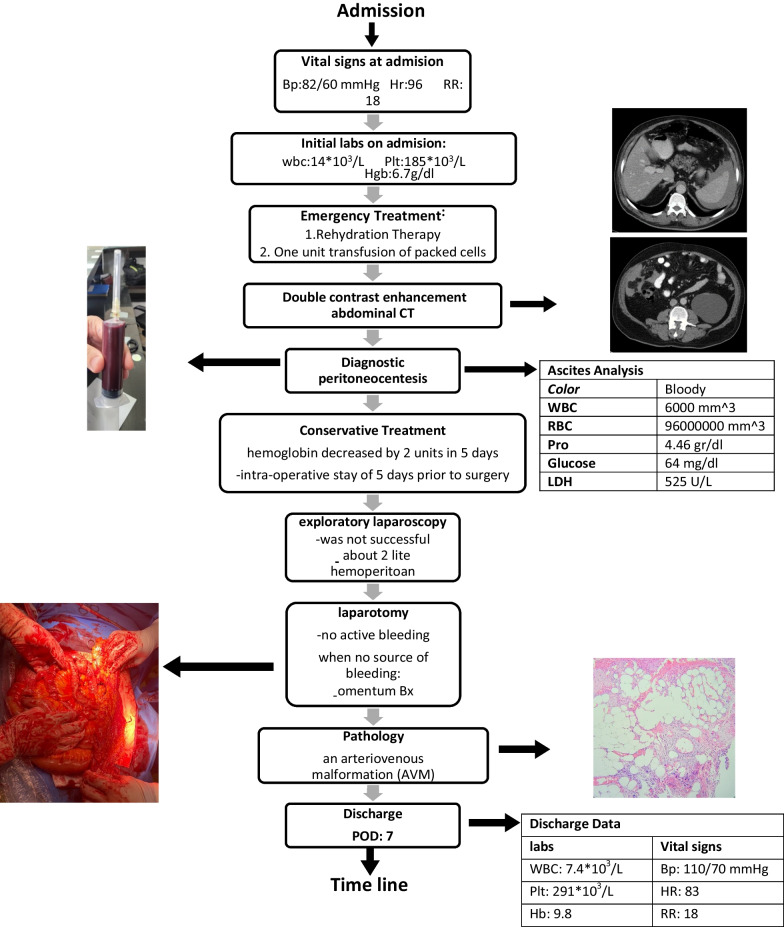
Timeline from emergency to follow-up in a patient who is suspected of AVM

A 65-year-old Iranian man arrived at the emergency department complaining of abdominal pain for 10 days with no history of recent trauma. Additionally, he suffered from diaphoresis and malaise. He had no significant previous medical history. Hematochezia, melena, anorexia, vomiting, nausea, or fever were not present. He was a non-smoker and non-alcoholic. Physical examination revealed 82/60 mmHg blood pressure, a temperature of 36.6 °C, and heart and respiratory rates of 96 beats and 18 breaths per minute, respectively. Examination of the abdomen showed symmetry without any scars. The abdominal assessment revealed only tenderness in the periumbilical region, and auscultation detected normal intestinal sounds. The focused assessment with sonography in trauma (FAST) examination yielded positive results. The complete blood count (CBC) reported white cell and platelet counts of 14 × 10^3^/L and 185 × 10^3^/L, respectively. Hemoglobin was 6.7 g/L at admission. One unit transfusion of packed cells was done. As soon as the patient was stable, a double contrast enhancement abdominal computer tomography (CT) was performed, which revealed massive hemoperitoneum. Through abdominal CT, we were unable to identify the source of bleeding. Hemoperitoneum was proved by ascitic tap ultrasound. During hospitalization, the hemoglobin value decreased by 2 units in 5 days. Subsequently, an exploratory laparoscopy was performed, searching for the responsible pathology. Approximately 2 L of blood was suctioned. But it was not successful. So, the surgical plan was changed to laparotomy. During laparotomy no active hemorrhage source was found. There was only a slight change (the lesion-like hematoma was 3 mm in size) in apparent omentum. Our decision was to take a biopsy of the omentum located on the greater omentum at the greater curve and send it for pathology analysis. Pathological assessment of the extracted tissue pointed to abnormally dilated blood vessels—an arteriovenous malformation (AVM) (Fig. 1). A 7-day hospital discharge followed the patient’s uneventful recovery. We performed follow-ups for the patient at the hospital outpatient department at 6 months intervals. The follow-up after 3 months showed that the patient had no signs of recurrence (Fig. 2). The timeline from emergency to follow-up is presented in Fig. 2.

## Review of the literature

PubMed and Scopus (2015–2022) databases were searched for case reports of idiopathic omental bleeding. Screening was conducted on all abstracts published in the English language. Data on patient characteristics, including age, diagnosis, and treatment, were extracted.

Out of the 12 articles, one of them contained three cases of idiopathic omental bleeding, which we have included in our study [5–16]. The relevant findings are summarized in Table 1. Patients ranged in age from 22 to 73 years old, including 10 males and 4 females. An abdominal CT scan, magnetic resonance imaging (MRI), an abdominocentesis, and a laparotomy were used in the diagnostic procedure. The patients underwent emergency surgery (*n* = 7) or transcatheter arterial embolization (TAE) (*n* = 4), and one patient underwent both. Five patients underwent omentectomy and three had ligation, all of which achieved hemostasis. One patient was managed non-operatively as a conservative case [5].

**Table 1:** Reports of idiopathic omental hemorrhage

First author	Year	Age (years)	Sex	Chief complaint	Past medical history	Anticoagulant use	Diagnostic procedure	Preoperative diagnosis	Therapy	Site of bleeding
Yuhang Zhou	2022	22	M	Abdominal pain (upper), dizziness, palpitations, amaurosis, cold sweats	Not mentioned	None	CT + contrast Abdominal paracentesis MRI + contrast Gastroscopy EUS	Intraperitoneal hemorrhage	Conservative	Upper (AVM)
Claire M. McClintock	2022	72	M	Death (with history of dyspepsia)	Metastatic prostatic carcinoma, IHD, HTN, CKD, and AF	NM	CT + contrast	Intraperitoneal hemorrhage	Autopsy	RUQ (pseudo/aneurysm)
Houcine Maghrebi	2022	68	M	Abdominal pain	HTN, HF	None	CT + contrast Angiography	Omental artery aneurysms	Angioembolization	Left gastroepiploic artery (aneurysms)
K Furukawa	2021	56	M	Abdominal pain (RUQ)	None	None	CT + contrast	Omental hemorrhage	Angioembolization	RUQ (gastroduodenal artery)
Shoryu Takayama	2021	53	F	Abdominal pain (RUQ)	Rheumatoid arthritis, appendicectomy cesarean section	None	CT + contrast	Omental hemorrhage	Laparoscopy partial omentectomy	RUQ
Heather K. Moriarty	2020	60	M	Faint, epigastric pain	Hemophilia B, Hepatitis C, previous intraabdominal idiopathic bleed (conservative)	None	CT angiography	Greater omental artery aneurysm	Angioembolization	Greater omental (upper)
Heather K. Moriarty	2020	37	M	Faint, abdominal pain	HTN, GERD	NM	CT + contrast	Omental hemorrhage	Laparotomy + bleeding site ligation	Greater omentum at the greater curve
Heather K. Moriarty	2020	69	F	Abdominal pain	Paroxysmal arterial fibrillation, HTN, cardiac failure, mitral valve repair	Apixaban	Angiography	Gastroduodenal artery aneurysm	Angioembolization	Gastroduodenal artery
J. Viñas	2020	66	M	Abdominal pain, decrease in consciousness, and hypotension	Bentall–De Bono surgery, hypertension, SARS-CoV-2 pneumonia	Enoxaparin	CT + contrast Angiography	Omental hemorrhage	Angioembolization Laparotomy partial omentectomy	Right gastroepiploic (pseudoaneurysm)
Yun-Xiao Lyu	2018	58	M	Abdominal pain (LUQ)	None	None	CT + contrast/abdominocentesis	Omental hemorrhage	Laparotomy partial omentectomy	LUQ (AVM)
Yen-Hung Wu	2017	35	M	Abdominal pain (LUQ)	None	None	CT + contrast	Omental hemorrhage	Laparotomy + bleeding site ligation	LUQ
Jiro Kimura	2016	29	M	Abdominal pain (LUQ)	None	None	CT + contrast	Omental hemorrhage	Laparotomy partial omentectomy	LUQ (right gastroepiploic)
Toshimitsu Hosotani	2016	38	F	Vomiting, diarrhea, abdominal pain (LLQ)	Appendectomy	None	CT + contrast TVS Culdocentesis	Omental hemorrhage	Laparoscopy partial omentectomy	LLQ
Zhang Zhong Tao	2015	73	F	Abdominal pain, abdominal discomfort, nausea, and vomiting	None	None	CXR, CT + contrast	Omental hemorrhage	Laparotomy + bleeding site ligation	Upper, lesser omentum

M male, F female, AVM arteriovenous malformations, CT computerized tomography, MRI magnetic resonance imaging, EUS endoscopic ultrasound, RUQ right upper quadrant, LUQ left upper quadrant, IHD ischemic heart disease, HTN hypertension, CKD Chronic kidney disease, AF atrial fibrillation, HF heart failure, GERD gastroesophageal reflux disease, NM not mention

## Discussion

Omental bleeding, with a mortality rate exceeding 30%, is a serious condition [17]. The mortality rate of 30% could be linked to the delayed diagnosis of most cases. It is worth mentioning that only a small number of cases were accurately diagnosed prior to treatment, which may have played a role in the elevated mortality rate.

FAST is frequently employed to expedite the prompt identification of life-threatening hemorrhage in patients. The majority of patients with positive FAST results require laparotomy [18].

Idiopathic omental bleeding requires aggressive treatment, regardless of its underlying cause. Omentectomy or ligation are routine surgical procedures for idiopathic omental hemorrhage. Most reported cases, however, involved emergency surgery. The surgical option is suitable for patients with persistent hypotension and unconfirmed diagnoses. The reason why surgery is often needed is that few cases are diagnosed correctly before treatment [12].

Vascular malformations (VMs) are treated with surgery and embolization. Embolization through endovascular means is less invasive and recommended in most cases. Life-threatening conditions can arise due to VMs because of their unpredictable clinical evolution and manifestations [19].

The cases presented in this manuscript are spontaneous, with no history of trauma, coagulopathy, or comorbidities, except for two [6, 7]. Abdominal pain was reported by all patients. LUQ, RUQ, and upper greater omental are the most common sites of bleeding.

Omental hemorrhage commonly presents in male patients with abdominal pain and occasionally with nausea, vomiting, or diarrhea. However, some of our included cases may not have had any other abdominal symptoms [6, 8–13].

Ultrasonography, computed tomography scan with contrast, chest x-ray [5], angiography [6], MRI with contrast, and paracentesis [5] may be useful to establish the diagnosis.

Optimal diagnostic and therapeutic evidence for spontaneous intraperitoneal hemorrhage remains controversial, based on the available literature. Through our case analysis and literature review, it has been observed that the prevailing diagnostic model for arteriovenous malformation primarily relies on the presence of positive FAST, abdominal pain, imaging evidence of fluid accumulation, and a reduction in hemoglobin levels, all of which collectively indicate the likelihood of AVM occurrence. CT angiography (CTA) may have false negative results, however, due to short acquisition times when bleeding is not obvious at time of scan [20]. It has been described that transcatheter arterial embolization is a definitive treatment [21].

A laparotomy or laparoscopy coupled with an omentectomy or a simple artery ligation is recommended. Laparoscopic surgery or transcatheter arterial embolization have been used more frequently in recent years as minimally invasive interventions [21, 22].

As described in the report, a patient presented to our hospital with sudden, non-specific abdominal pain, which was diagnosed with idiopathic spontaneous intraperitoneal hemorrhage caused by a vascular malformation in the omentum.

## Conclusion

A spontaneous intraabdominal hemorrhage without any antecedent trauma should begin with volume resuscitation to stabilize the patient’s circulatory parameters. Coagulation studies and platelet function analysis should be considered. Hemorrhages of the omentum, however, are infrequent, and patients’ conditions are often unstable. In cases where no source of bleeding is found, a high index of suspicion should be kept. Presence of positive FAST, abdominal pain, imaging evidence of fluid accumulation, and a reduction in hemoglobin levels collectively indicate the likelihood of AVM occurrence. The venous hypertension, which can be caused by factors such as consuming food or engaging in extensive sports, can lead to reduced perfusion pressure in the surrounding tissues. For both unconfirmed and definitive diagnoses and treatments, emergency surgery is recommended. To rule out underlying malignancy or vascular disease, omentectomy is preferred to ligation or transcatheter arterial embolization. Rebleeding should be eliminated from these patients by omentectomy as a definitive therapy.

## Data Availability

Not applicable.

## Abbreviations

FAST: Focused assessment with sonography in trauma
CBC: Complete blood count
CT: Computer tomography
TAE: Transcatheter arterial embolization
AVM: Arteriovenous malformation
MRI: Magnetic resonance imaging
VMs: Vascular malformations
CTA: CT angiography
